# Signaling Response to Transient Redox Stress in Human Isolated T Cells: Molecular Sensor Role of Syk Kinase and Functional Involvement of IL2 Receptor and L-Selectine

**DOI:** 10.3390/s20020466

**Published:** 2020-01-14

**Authors:** Christian Secchi, Marco Orecchioni, Marissa Carta, Francesco Galimi, Francesco Turrini, Antonella Pantaleo

**Affiliations:** 1 Department of Obstetrics, Gynecology, and Reproductive Sciences, University of California, San Diego, School of Medicine, La Jolla, CA 92093, USA; 2 Department of Biomedical Sciences, University of Sassari, I-07100 Sassari, Italy; marissacarta29@gmail.com (M.C.); fgalimi@uniss.it (F.G.); 3 Istituto Nazionale Biostrutture e Biosistemi, University of Sassari, I-07100 Sassari, Italy; 4 La Jolla Institute of Immunology, La Jolla, CA 92093, USA; morecchioni@lji.org; 5 Department of Chemistry and Pharmacy, University of Sassari, I-07100 Sassari, Italy; 6 Department of Oncology, University of Turin, I-10126 Turin, Italy; francesco.turrini@unito.it

**Keywords:** biomarkers, T cells, Syk, redox signaling, Tyr phosphorylation, redox stress

## Abstract

Reactive oxygen species (ROS) are central effectors of inflammation and play a key role in cell signaling. Previous reports have described an association between oxidative events and the modulation of innate immunity. However, the role of redox signaling in adaptive immunity is still not well understood. This work is based on a novel investigation of diamide, a specific oxidant of sulfhydryl groups, and it is the first performed in purified T cell tyrosine phosphorylation signaling. Our data show that ex vivo T cells respond to –SH group oxidation with a distinctive tyrosine phosphorylation response and that these events elicit specific cellular responses. The expression of two essential T-cell receptors, CD25 and CD62L, and T-cell cytokine release is also affected in a specific way. Experiments with Syk inhibitors indicate a major contribution of this kinase in these phenomena. This pilot work confirms the presence of crosstalk between oxidation of cysteine residues and tyrosine phosphorylation changes, resulting in a series of functional events in freshly isolated T cells. Our experiments show a novel role of Syk inhibitors in applying their anti-inflammatory action through the inhibition of a ROS-generated reaction.

## 1. Introduction

ROS have been known as markers of cellular stress for long time. However, they can act as second messengers in the intracellular signaling and can potentially activate and control a multiplicity of function in different cell type [1,2,3,4,5]. Numerous reports have focused on the redox regulation of the immune system. Particularly, thiol groups oxidation appears to be strongly influenced by reactive oxygen species (ROS) generating a cellular response [6,7,8].

Thus, ROS need to interact with molecular sensors, usually cysteine residues, that through the formation of a reversible disulphide bond sense the redox changes and activate intracellular signalling pathways through the formation of a reversible disulfide bond [9,10]. The sulfenyl moiety is present in cysteine residues and can form a disulfide bond with another thiol moiety. The phenomenon is transient since both sulfenyl moiety and disulfide bond can be reduced by various endogenous antioxidants in cells. For this particular transient and reversible nature, the cysteine oxidation is now considered as a post-translational modification [11,12].

This assumption is validated by the fact that a large number of proteins involved in cell signaling pathways contains critical thiol groups whose oxidation alters their activity [13,14]. Nevertheless, a decent number of works have been recently published on the mechanisms of redox regulation in different hemopoietic and immune models [15,16,17].

Studies on erythrocyte membrane stability [18,19,20] revealed that oxidation induces a phosphorylation response that specifically involves 2 tyrosine residues located in the cytoplasmic domain of band 3 protein. It has been also described how several hemolytic disorders are related to the increase in membranes with oxidative phenomena, such as β-thalassemia [21,22], G6PD deficiency [23,24], sickle-cell anemia [25,26,27], malaria-infected RBCs [28,29,30], and also Alzheimer’s disease [31], Parkinson’s disease [32], Crohn’s disease [33,34], and cardiovascular diseases [35,36].

Also, various immune cell populations including T and NK cell subsets have been analyzed in regard to their sensitivity toward ROS. In particular, T cells may be considered one of the important targets for investigations on oxidative signaling in pathological condition [37,38]. Autoimmune diseases with a chronic inflammation component are characterized by a strong production of ROS and leukocytes recruitment [39,40,41]. Many reports have shown that oxidative stress in this kind of cells is not only associated with cancer but also represents an important immune escape mechanism in autoimmune diseases associated with chronic inflammation [42,43]. Systemic lupus erythematosus (SLE) [44], rheumatoid arthritis [45], and allergies [46] are among the most notable examples of immune alterations related to oxidative conditions in which T cells are involved.

The complex effects of ROS on T cells have been studied with numerous experimental models, that had included H_2_O_2_ exposure or co-culture with monocytes/macrophages [47,48,49,50]. Tumor-associated macrophages (used as ROS source) and H_2_O_2_ or diamide were co-cultured in T cells causing a decreased CD3 zeta chain expression of the TCR [50,51]. Moreover, T cells by macrophages isolated from metastatic lymph nodes from patients with malignant melanoma or by LPS-stimulated monocytes showed a CD16 decline [47]. H_2_O_2_ treated Jurkat T cells suggested the connection of redox-effectors in several cell functions as protein degradation, metabolism, cytoskeleton regulation and signaling [52]. A report in 2009 has showed that Treg cells differ from other T-cell subclasses. Indeed, treatment with H_2_O_2_ high concentrations, usually highly noxious for CD4 + T cells, did not affect Treg cells demonstrating a specific resistance to ROS-induced cell death [53]. Also, ROS function as mediators for recruiting leukocytes to wounded areas. Src family kinase (SFK), Lyn was revealed to react to ROS. Yoo et al. discovered that this kinase is elicited by wound-derived H_2_O_2_ and induce leukocyte chemotactic movement to the wounds. Lyn appears to be the direct target by H_2_O_2_ oxidation of Cys^466^ [39,54,55]. These studies show that it is useful to investigate the kinases and phosphatases and their role as biosensors in oxidative conditions [56]. Published reports are focused on the notion that the major targets of ROS involve protein tyrosine phosphatases (PTPs) and the oxidation of their cys residues provoking their activity inhibition [57,58]. One of this study showed that SH2 domain protein tyrosine phospatase-2 (SHP-2) is affected by ROS in platelets leading to its inactivation and direct activation by Tyr phosphorylation of the spleen tyrosine kinase (Syk) and other tyrosine kinases of TCR. Nevertheless, we published our findings on Jurkat T cells which respond to –SH group oxidation with specific tyrosine phosphorylation events. Experiments with spleen tyrosine kinase (Syk) inhibitors showed upstream participation of Syk in these responses [59].

**Figure 1:**
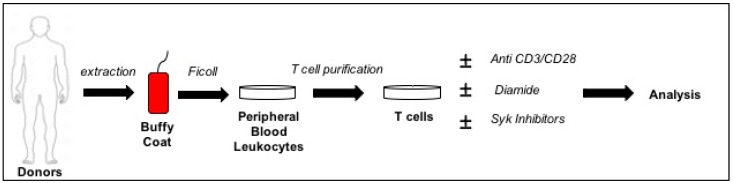
General experimental plan of the study. Buffy coat with peripheral blood leukocytes were extracted from healthy donors. T cells were obtained by Ficoll/Histopaque centrifugation and subsequent column purification. They were left 24 h before to be treated with activators/diamide/syk inhibitors.

Here we report for the first time an ex vivo study on isolated T cells from healthy donors aimed to monitor the phosphorylation response to a temporary redox stress induced by diamide in the presence or absence of Syk Inhibitors (Figure 1). We explicitly performed experiments using this oxidant reagent as oxidant agent in order to evade the complexity of other models (e.g., co-cultures) or applying H_2_O_2_ since it is quickly degraded and provokes radical formation reacting in an unpredictable way. On the contrary, diamide specifically and reversibly oxidizes –SH groups [60,61]. We also investigated the functional role of the Syk kinase and the expression of the essential T cell CD25 and CD62L receptors with the main T cell cytokines in our ex vivo *model*. Our findings also showed that Syk inhibitors can stop the response initiated by ROS implying that their anti-inflammatory action involves adaptive immunity via T-cell modulation.

## 2. Materials and Methods

### 2.1. Human T Cells from Donors

Experiments were performed isolating T lymphocytes from male healthy donor buffy coat. This study was conducted in accordance with Good Clinical Practice guidelines and the Declaration of Helsinki. Peripheral blood leucocytes (PBLs) were purified by density gradient centrifugation, using the separation medium HISTOPAQUE 1077 (Sigma-Aldrich, St. Louis, MO, USA) according to Böyum method. T cells were isolated using high affinity CD4+ T-cell enrichment columns (R&D Systems, Minneapolis, MN, USA). Subjects have given their written informed consent. The study protocol has been approved by the Research Institute’s Committee of University of Sassari on human research. After isolation, T cells viability was evaluated with trypan blue assay and cells were maintained in RPMI 1640 supplemented with 10% (v/v) heat-inactivated FBS, 20 mM HEPES, 10 mL/L penicillin/streptomycin at 37 °C. Cell suspensions were left for at least 12 h at 37 °C to allow recovery from stress because of the purification procedures. For our investigations, 8 × 10^5^/mL T cells were resuspended at the in fresh medium.

### 2.2. T Lymphocytes Activation

T lymphocytes from blood donors were activated with incubation of anti-CD3 (4 μg/mL) and anti CD28 (4 μg/mL). All the T cell samples were activated in the present study. We specified in the legends when they were not activated.

### 2.3. Syk Inhibitors

Pre-incubation with 5 µM Syk inhibitors II (Calbiochem, San Diego, CA, USA) was performed for 1 h at 37 °C previous oxidant treatments. Phosphate buffer saline (PBS) wash was applied prior to the oxidant treatment.

### 2.4. Oxidant Agent

Diamide is an exclusive –SH group oxidant agent [43]. T cell transient oxidation was induced by exposure with Diamide (Sigma-Aldrich, St. Louis, MO, USA) that was first tested in dose response and 0.3 mM concentration was chosen for our experiments. Viability of the cells was examined by trypan blue dye exclusion test (Sigma-Aldrich, St. Louis, MO, USA). Time course of the incubated T Cells were performed at 37 °C. After treatment, cells were washed four times with PBS (150× *g*, 10 min).

### 2.5. MTT Reduction Test

0.3 mM diamide in RPMI 1640 was administered at distinctive times to the T cell samples in 96-well plate (Corning, NY, USA) in order to establish reduction activity. Next, 5 mg/mL Thiazolyl blue tetrazolium bromide (MTT, Sigma-Aldrich, St. Louis, MO, USA) was incubated for 4 h. After the incubation, the medium was replaced with 0.3 mL MTT solvent solution (4 mM HCl, 0.1% Nondet P-40 in isopropanol) in order to solubilize the converted dye. Microplate reader (Biorad, Hercules, CA, USA) was used to measure the absorbance at 570 nm. The reduction activity is showed as percentage of the compared control.

### 2.6. Electrophoresis and Immunoblotting

DC protein assay (Biorad, Hercules, CA, USA) was performed to the samples after the experimental incubations in order to quantify the total protein amount. Samples were then solubilized in Laemmli buffer with 2% betamercaptoethanol and heated at 95 °C (5 min). Solubilized protein samples were loaded (30 μg) and run in SDS-PAGE. Next, the proteins were transferred to nitrocellulose membranes by western blot analysis. Membranes were blocked in PBS/Tween. 1:2000 Anti-phosphotyrosine antibody (Santa Cruz, CA, USA) was used. Secondary rabbit antibody conjugated to infrared fluorescent dyes excitable at 800 nm (IRDye 800CW, Licor, Lincoln, NE, USA) was incubated at 1:25,000 for 1 h and then washed. In order to visualize the antigens, 800 nm laser scanner (Odyssey, Licor, Lincoln, NE, USA) was used. Quantitative analysis of proteins on the membranes was performed by ImageJ (USA).

### 2.7. Membrane Receptor and Intracellular Analysis

Flow analysis was carried out to quantity expression of CD25 (IL-2 receptor) and CD62L (L-Selectine) surface receptors. Antibodies for each marker were purchased from BD Biosciences (Mountain View, CA, USA) and incubated for the analysis with FACS CANTO (BD Biosciences, Mountain View, CA, USA). FlowJo software (BD Biosciences, Mountain View, CA, USA) was used for the analysis of the results. To analyze the oxidation-related DNA damage status, CD 4+ T cells were isolated and treated or untreated as previously described with Syk Inhibitors for 1 h before the incubation with diamide (0.3 mM) started. Untreated cells were used as a control. After the incubation with diamide, cells were harvested and washed twice with PBS. To perform the intracellular staining, cells were incubated with the 1X Fix/Perm buffer (eBioscience, San Diego, CA, USA) for 30 min at RT, washed with PBS, resuspended in 1X permeabilization buffer (eBioscience, San Diego, CA, USA), and incubated with the mouse anti-human p-Histone H2A.X antibody (1:200, Santa Cruz Biotechnology, Dallas, TX, USA) for 20 min and then incubated with the secondary antibody FITC-goat anti-mouse IgG (1:200 Clone: Poly4053, Biolegend, San Diego, CA, USA) for 20 min at RT in the dark. After incubation, cells where washed twice with PBS and analyzed by flow cytometry FACS CANTO (BD Biosciences, Mountain View, CA, USA). Experimental data analysis was performed with FlowJo software (BD Biosciences, Mountain View, CA, USA).

### 2.8. Cytokines Analysis

After the 0.3 mM diamide and Syk inhibitors incubation supernatants were stored for analysis. Cytokines bead array (CBA) human, Th1/Th2, cytokine kit (BD Biosciences, Mountain View, CA, USA) following the manufacturer protocol was used in order to quantify IL2, IFNγ, and TNFα. The experiments were carried out using the FACS CANTO (BD Biosciences, Mountain View, CA, USA). Experimental data analysis was performed with the FCAP Array software, version 3.0 (BD Biosciences, Mountain View, CA, USA).

### 2.9. Statistical Analysis

GraphPad Prism software was used to perform all the statistical analysis and significance was measured by unpaired *t*-test and Mann-Whitney *U* test and the two-way or one-way analysis of variance (ANOVA). A *p* value less than 0.05 was considered significant.

## 3. Results

### 3.1. Diamide Does Not Affect T Cell Viability

**Figure 2:**
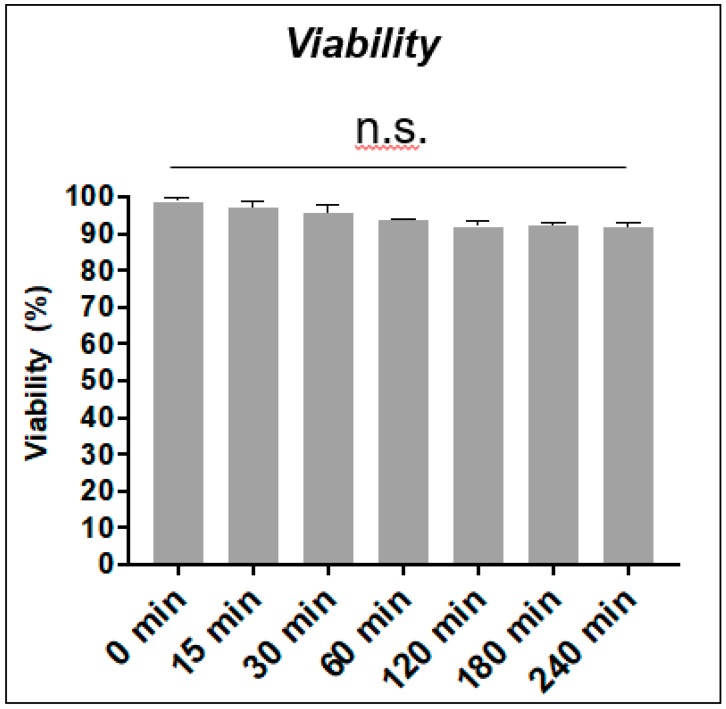
Trypan blue viability assay. Trypan blue assay of isolated T cells at different time points (from 15 min to 240 min) of 0.3 mM diamide oxidation. Viability is expressed as percent of total cell number (%). *p* value of the viability time points was found to be statistically non-significant (N = 3).

Diamide is a specific –SH group oxidant that, forming intermediate thiyl radicals, mainly generates reversible disulfide bonds. Its concentration was chosen to observe those changes avoiding cell toxicity. Experiments with purified human T cells were performed in order to clarify the phenomena observed with Jurkat T cell line [59]. After purification from healthy donor blood, T cells were treated with 0.3 mM diamide (Figure 1). Figure 2 shows the treatment does not affect cell viability over time.

### 3.2. Diamide Oxidation Affects the Tyr Phosphorylation of T Cell Proteins

**Figure 3:**
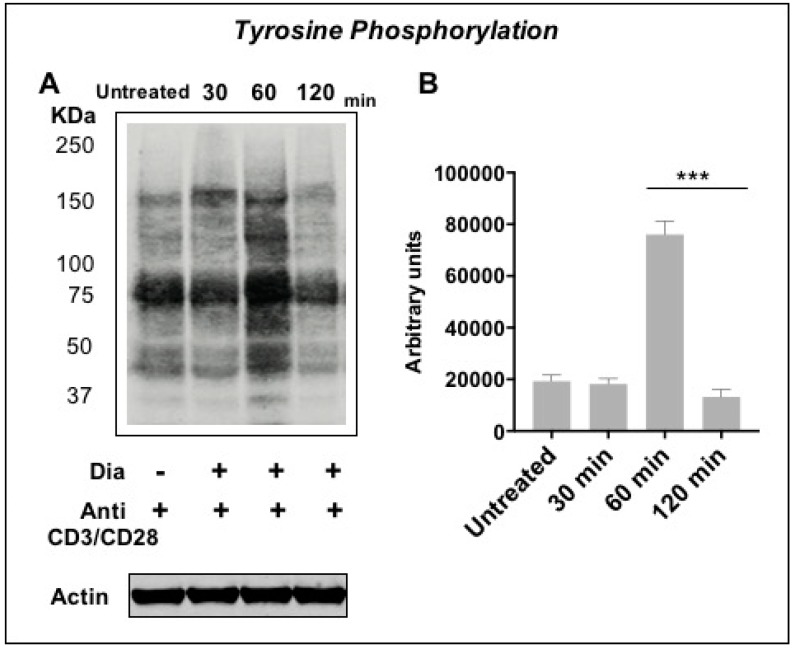
T cells tyrosine phosphorylation under oxidative condition. (**A**) Western blots of anti-phosphotyrosine T cells total proteins treated with 0.3 mM diamide. Beta-actin loading control is showed. (**B**) Quantification of tyrosine phosphorylation levels was performed by IR fluorescence detection (Odyssey, Licor, USA). *** (*p* < 0.001) indicate the incubation time that determines a statistically significant change between samples measured by student *T*-test (N = 3).

As described previously [59], we found a molecular and functional relationship between SH group oxidation and Tyr phosphorylation in Jurkat cells. We here investigated if this mechanism is maintained in purified primary T cells. Studies on CD4+ T cells were performed under activation conditions. After stimulation with AntiCD3/AntiCD28 antibodies, T cells were treated with diamide. Tyrosine phosphorylation changes at different incubation times (15, 30, 60, and 120 min) were measured. We observed a phosphorylation peak after 60 min incubation (Figure 3A,B) that decreased at 120 min. Of note, those changes are similar to the response observed in Jurkat cells and erythrocytes, which display the lowest GSH levels after 30 min and restore basal GSH levels within 60 min [23,59].

### 3.3. Oxidation and Tyr Phosphorylation Have Same Time Course

**Figure 4:**
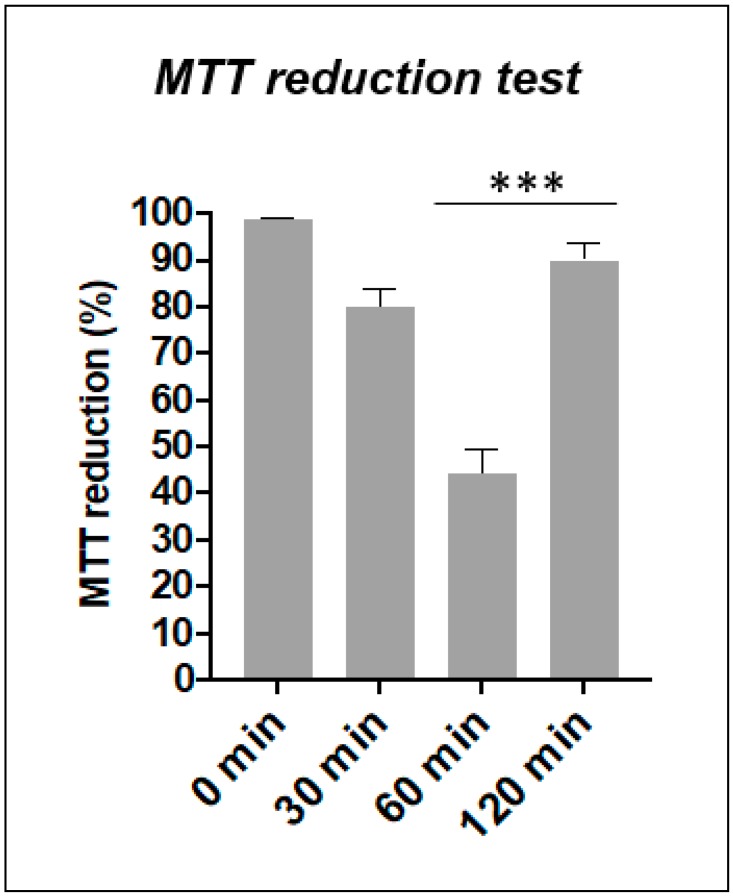
MTT reduction test. MTT reduction test of T cells treated with 0.3 mM diamide. Reduction activity is expressed as percent of untreated cells (N = 3). *** (*p* < 0.001) indicates the incubation time that determines a statistically significant change between samples measured by student *T*-test.

The level of oxidation in activated CD4+ T cells was quantified by MTT test applying the same time course (from 0 to 120 min); in order to confirm that oxidation is related to the observed phosphorylation phenomena. Primary T cells treated with 0.3 mM diamide present highest level of oxidation at 60 min (Figure 4). Again, the transient nature of these changes is shown by the following increase of MTT reduction signal at 120 min.

### 3.4. Syk is Involved in Tyrosine Phosphorylation Changes in Activated T Cells under Oxidative Condition

**Figure 5:**
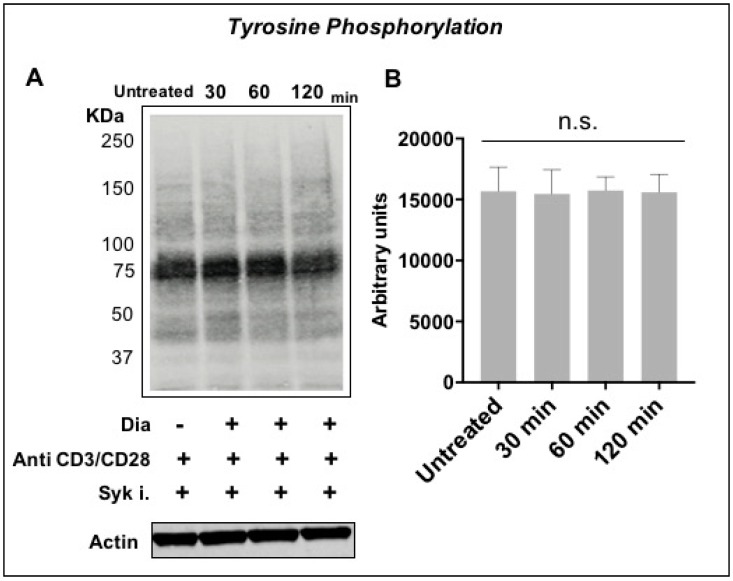
Role of Syk in T cells under oxidative condition. (**A**) Immunoblot of T cells treated with 0.3 mM diamide in the presence of Syk inhibitors 5 μM (Syk i.), at different incubation times (0, 30, 60, 120 min). Beta-actin loading control is showed. (**B**) Quantification of tyrosine phosphorylation levels was performed by IR fluorescence detection (Odyssey, Licor, Lincoln, NE, USA) of antiphosphotyrosine Western blots and expressed as fluorescence arbitrary units (N = 3).

It is well-known that Syk is expressed in primary T cells and plays a key role as a stress sensor. Activated T cells were pre-treated with Syk inhibitors and then exposed to oxidative conditions with 0.3 mM diamide. Figure 5A,B shows the comparison between the patterns of T cell proteins incubated. This experiment showed that Syk appears to be involved in tyrosine phosphorylation cascade since pre-incubation with Syk inhibitors strongly inhibits the tyrosine phosphorylation signal. Interestingly, detection of histone H2AX phosphorylation on Ser-139 that is known as an indicator of DNA damage (DNA double-strand breaks) [62,63,64] showed that the peak of the DNA damage was at 1-h diamide exposure coherently with our phosphorylation data and then decrease. Surprisingly, Syk inhibitors showed a protective action to the DNA breakage (Figure S1 (Supplementary Materials)).

### 3.5. CD25 and CD62L are Affected by Diamide Treatment and Syk Inhibition in Purified T Cells

Next, we sought to investigate the functional activity between SH group oxidation and Syk inhibition through flow cytometry quantification analysis of membrane marker (CD69, CD25, and CD62L) expression (gating strategy in Figure S2 (Supplementary Materials)). After the treatment, CD69 was not affected by the treatment (data not shown). Moreover, T cells after isolation had a high rate of purity (99%) as showed in Supplemental Figure S2.

### 3.6. Surface Expression of IL-2R Receptor

**Figure 6:**
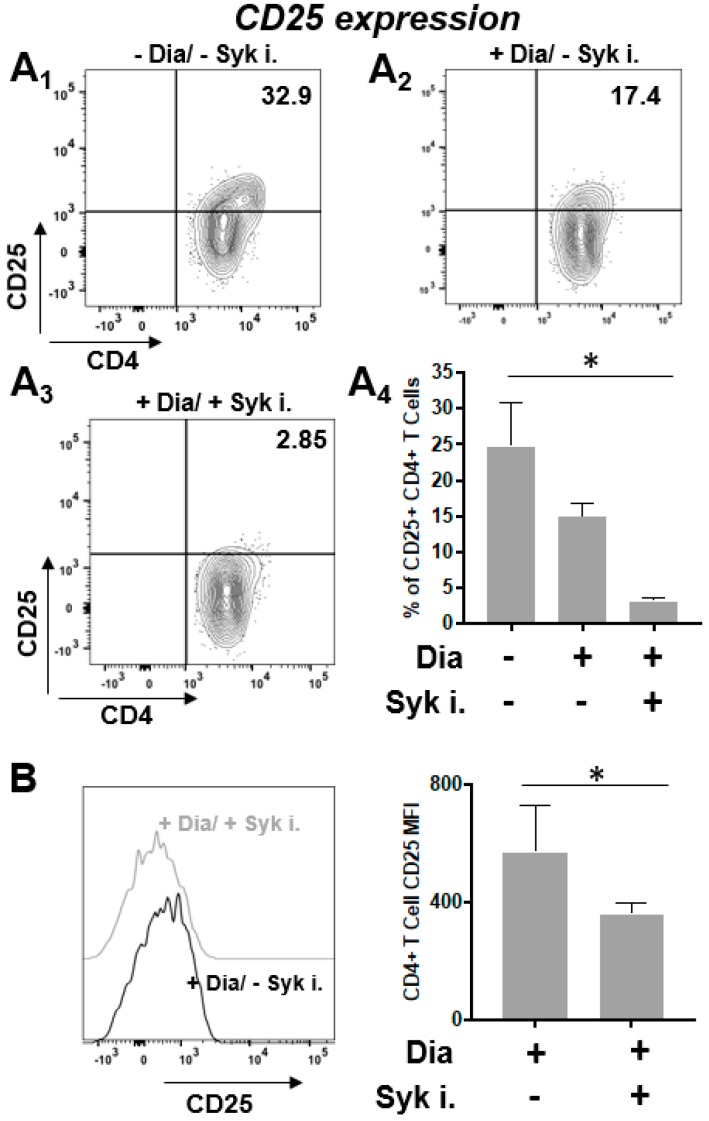
Expression of T cells CD25 receptor after treatment with diamide and Syk inhibitors. Cells exposed to 0.3 mM diamide with/out 5 μM Syk inhibitors (Syk i) at 60 minutes incubation time. Samples were incubated surface-CD25 and analyzed by Flow cytometry (N = 3). (**A1**) Anti-human CD25 flow representative density plot of untreated cells. (**A2**) Anti-human CD25 flow representative density plot of T cells exposed to 0.3 mM diamide. (**A3**) Anti-human CD25 flow representative density plot of T cells exposed to 0.3 mM diamide and incubated with Syk inhibitor. Medians are showed. *p* values which were statistically significant are shown (* *p* < 0.05). Values are plotted as mean ± error standard (**A4**). Data are the percentage of total cell population (%). (**B**) Samples were incubated with human surface anti-CD25 and analyzed by Flow cytometry. Comparison of MFIs between diamide-treated T cell samples in presence versus absence of Syk inhibitors incubation.

Treatment with 0.3 mM diamide triggered a decrease of CD25 expression in the activated T cells at 1 h of exposure (see Figure 6A1,A2). Moreover, the diamide treatment in the non-activated cells induced a slight CD25 increase (Figure S3 (Supplementary Materials)). The incubation of Syk inhibitors had a stronger effect since it synergistically reduced the percentage of cells (Figure 6A3,A4) and the mean fluorescence intensity signal of CD25 (Figure 6B) at the same time point in presence of diamide (see control without diamide in Figure S4 (Supplementary Materials)).

### 3.7. Surface Expression of L-Selectin

**Figure 7:**
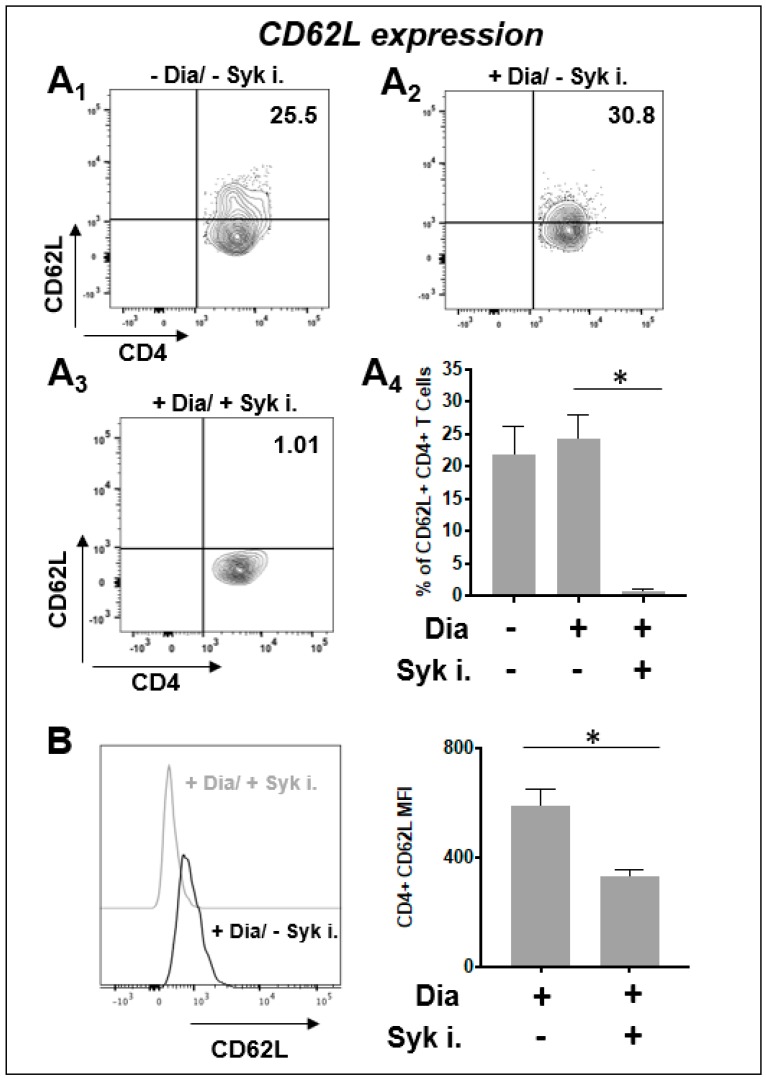
Expression of T cells CD62L receptor after treatment with diamide and Syk inhibitors. Cells exposed to 0.3 mM diamide with/out 5 μM Syk inhibitors (Syk i) at 60 minutes incubation time. Samples were incubated surface-CD62L and analyzed by Flow cytometry (N = 3). (**A1**) Anti-human CD62L flow representative density plot of untreated cells. (**A2**) Anti-human CD62L flow representative density plot of T cells exposed to 0.3 mM diamide. (**A3**) Anti-human CD62L flow representative density plot of T cells exposed to 0.3 mM diamide and incubated with Syk inhibitor. Medians are showed. *p* values which were statistically significant are shown (* *p* < 0.05). Values are plotted as mean ± error standard (**A4**). Data are the percentage of total cell population (%). (**B**) Samples were incubated with human surface anti-CD62L and analyzed by Flow cytometry. Comparison of MFIs between diamide-treated T cell samples in presence versus absence of Syk inhibitors incubation.

Activated T cells showed high expression of CD62L both in control and 1-h oxidation samples (Figure 7A1,A2). Surprisingly Syk inhibitors had the effect to dramatically decrease the expression of L-Selectin (Figure 7A3,A4,B) (see control without diamide in Figure S4 (Supplementary Materials)).

### 3.8. SH Group Oxidation and Syk Inhibition Activity on Cytokine Release

We next measured by FACS analysis a specific set of cytokines (Figure S5, Supplementary Materials). Diamide exposure provoked an increased release trend of IFNγ, IL2 and TNF. Syk inhibition strongly reduced the effects of diamide in basal and 60 min samples (see 60 min samples in Figure S5, Supplementary Materials).

## 4. Discussion

ROS have long been identified as markers of cellular stress [65]. Oxidant high concentrations in inflammation outbreaks and their possible functions in immune cells are relevant to better understand the physiopathology of various disorders. Still studies on autoimmune diseases associated with chronic inflammation and characterized by a strong production of ROS and T cell recruitment can be considered a good model to better understand the role of oxidation in diseases [37]. Indeed, ROS play a key role in the onset of rheumatoid arthritis (RA) and systemic lupus erythematosus (SLE) [44,66]. However, it has been nowadays acknowledged that ROS are not only associated with stress/pathological conditions but also the temporary and low amounts of ROS are important contributors to physiological signaling pathways implicated in cell growth, controlled cell death, migration, and T cell activation [38,67,68]. T lymphocytes can sense stress condition [69] and are subject to oxidative stress in the inflammation occurring under different pathological states [70,71], but recently evidences indicate the involvement of ROS in T-cell activation and proliferation [72,73]. In this line, the role of redox sensitive NFkB and Nrf2 transcription factors in T cell activation has been already demonstrated [74,75]. However, limited information is still available on the regulatory mechanisms of ROS in T cell. Indeed, the mechanisms of redox signaling are incompletely understood, beyond the inhibition of some protein tyrosine phosphatases (PTPs) containing a regulatory cysteine residue in their catalytic domain [58,76].

ROS need to interact with protein sensors to generate a cellular response. Usually they are cysteine residues-based effectors that can sense the redox changes and trigger intracellular signalling pathways through the formation of a reversible disulphide bridge [77]. The regulatory cysteine residues of some PTPs display a relatively low reactivity (1000-fold lower than GSH) to oxidants [58]. PTPs should therefore be inactivated only at very high concentrations of ROS since the occurrence of high intracellular concentration of GSH. Consequently, these mechanisms could involve kinases that are required to elicit specific phosphorylation pathways. Since protein tyrosine phosphorylation is nearly invariably involved in the T-cell regulatory response to various stimuli [78], we performed a study on the protein Tyr phosphorylation changes after treating primary T cells with a reversible thiol group reagent at non-cytotoxic concentrations (Figure 1 and Figure 2).

**Figure 8:**
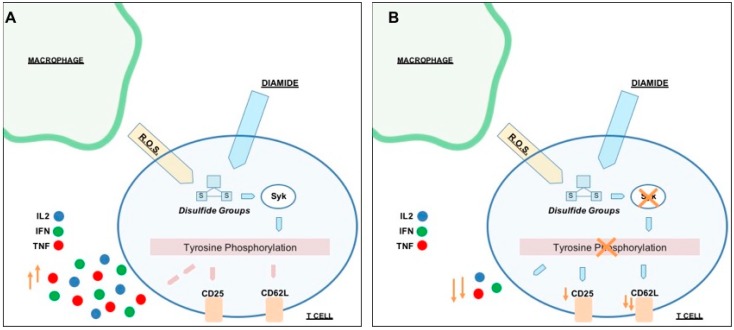
Model of the cysteine redox regulation of the Tyr phosphorylation in human purified T cells. (**A**) Different sources, such as macrophages, can produce oxidative species. Oxidation of thiol groups may directly induce the Syk activation. This kinase is essential in the consequent phosphorylation and functional phenomena. These molecular events involve several different effectors and generate a cellular response on the T cells receptors. (**B**) Syk inhibition blocks the ROS-trigged phosphorylation flow and further affected CD25 and CD62L receptors and cytokine liberation.

We previously published that the Jurkat T cell line can sense the cysteine-specific transient oxidation generating a phosphorylation cascade where the Syk kinase has a pivotal role [59]. Syk has been demonstrated to be reversibly activated by –SH reagents B and T cells [59,79]. It should be also noticed that the accessibility of the cysteine residues located in the catalytic site of PTPs displays a lower reactivity than GSH, therefore ROS should be efficiently buffered by GSH before inhibiting PTPs [6]. Therefore, more sensitive ROS sensors like the Syk kinase should be required to trigger intracellular signaling. In this work we have observed for the first time in fresh isolated CD4+ T cells from healthy donors that the reversible –SH group oxidation induced a transient increase of protein Tyr phosphorylation, providing an indication in which the oxidation of effector cysteine residues may induce a specific cell response. As mentioned above, our Secchi et al. investigations [59] were based on Jurkat T cells, that are definitely a useful tool for immunological explorations but may have the limit to not represent what actually occur from a physiological view point. Therefore, the main innovation of our investigation is showing the crosstalk between the two posttranslational modifications (Tyr phosphorylation and Cys oxidation) and the ROS-biosensor Syk role are physiological events since present and active *in human freshly-purified human T cells* (Figure S2 (Supplementary Materials)). This is quite important from a biological and clinical point of view because it defines that redox regulation by Cys oxidation as a biological phenomenon that generally occurs in CD4+ T cells in physiological and pathological condition. Activated T cells were treated with diamide, a specific –SH oxidant reagent, and their tyrosine signal reached at peak at 60 min and then decreased (Figure 3). MTT reduction assay validates the parallelism of these molecular events where the treatment of diamide showed a temporary decrease of the metabolic activity (almost half reductive activity is compromised) and tyrosine phosphorylation peak. Interestingly, those changes were reversed by Syk inhibitors (Figure 4 and Figure 5 and Figure S4 (Supplementary Materials)). Inhibitors of Syk kinase have been developed over the last fifteen years able to block lymphocyte functions with possible applications in the treatment of several immune diseases [80]. They were described as drugs against the activation of other effectors such as JAK and MAPK [81]. A molecule in this class is the fostamatinib (R788); prodrug of R406 that we used in our investigation. To date, many clinical trials on R788 have been completed and some have already evaluated its safety and efficacy for the RA treatment. The trials denoted that fostamatinib is an effective therapeutic medicine administered to patients with over 24 weeks [82]. Our experiments showed a central role of Syk kinase in the T-cell response to redox stress indicating it has a key role upstream of the Tyr phosphorylation cascade. Interestingly and in line with our findings, Syk inhibition showed a protective action to the ROS induced DNA damage indicating its essential role to the signaling triggered by thiol oxidation (Figure S1, Supplementary Materials). Next, we performed a further investigation on a set of T-cell receptor and cytokines. The study indicated that specific functions respond differently to redox stress. Interestingly diamide oxidation affected the IL2 receptor (CD25) but not the L-Selectin (CD62L) (Figure 6A and Figure 7A). The CD25 downregulation may be due to the pleiotropic effect of the redox signaling: it is important to stress that the IL2 cytokine secretion is known to be decreased by oxidation [83,84] therefore it is plausible that concomitant decline in IL-2 secretion indirectly downregulated CD25 by the loss of the typical self-stimulatory IL2-IL2R action in T cells [85]. On the other hand, SH groups oxidation and Syk inhibition lack of effects on CD69 expression. We also observed efficiency of Syk inhibitors in turning down both receptors in a significant way (Figure 6B and Figure 7B). In particular CD62L was almost blocked (Figure 7A4). L-Selectin expression on naive CD4+ T cells is required for their efficient recirculation and compartmentalization between blood and lymph nodes. This data provides new insights into the mechanisms underlying Syk inhibitors anti-inflammatory activity since its action in decreasing L-selectin expression may have direct consequences on the T-cell recruitment and transmigration through the endothelial layer into tissues in early stages of inflammatory processes. Moreover TNF, IFNγ, and IL2 cytokines analysis (Figure S5, Supplementary Materials) showed a positive trend in correspondence of the maximal –SH group oxidation and Tyr phosphorylation responses. This cytokine trend disappeared in presence of Syk inhibition. In conclusion this work shows for the first time the reversible oxidation of sulfidryl groups induces a transient and very complex protein Tyr phosphorylation response in purified human T cells, as described in Figure 8A. This response may be mediated by a direct activation of Syk kinase and/or the known capability of ROS to inhibit PTPs. Syk inhibition efficiently reverted the phosphorylation response and provoked specific cellular changes establishing a possible causal linkage (Figure 8B). For the functional interpretation of the results, it should be also pointed out that our study has been conducted on purified T cells from blood donors, but the effects of ROS may be largely modulated by additional regulatory signals occurring in a variety of physiological and pathological situations that accompany the production of ROS.

## Supplementary Materials

The following are available online at https://www.mdpi.com/1424-8220/20/2/466/s1, Figure S1: Detection by FACS of histone H2AX phosphorylation on Ser-139; Figure S2: Gating strategy; Figure S3: Expression of non-activated T cells CD25 receptor after treatment with diamide; Figure S4: CD25 and CD62L surface receptor expression of T cells treated with Syk inhibitors; Figure S5: TNFα, IL2, and IFNγ cytokines released by T cells.

## Abbreviations

BSA	bovine serum albumin
CD16	cluster of differentiation 16
CD25	cluster of differentiation 25
CD3	cluster of differentiation 3
CD62L	L-selectin
Cys	cysteine
DIA	diamide
DMSO	dimethyl sulfoxide
FA	fluoroacetic acid
FACS	fluorescence-activated cell sorting
FBS	fetal bovine serum
G6PD	glucose-6-phosphate dehydrogenase
GAPDH	glyceraldehyde 3-phosphate dehydrogenase
GSH	glutathione
H2O2	hydrogen peroxide
IL2	interleukin 2
IL-2R	interleukin 2 Receptor
JAK	janus kinase
STAT	signal transducer and activator of transcription
JNK/ERK	c-jun N-terminal kinase/extracellular-signal-regulated kinase
JUNK	c-jun N-terminal kinases
LPS	lipopolysaccharide
Lyn	lyrosine-protein kinase
MHC	II major histocompatibility complex II
MTT	thiazolyl blue tetrazolium bromide
NF-KB	nuclear factor kappa-light-chain-enhancer of activated B cells
Nrf2	NF-E2-related factor 2
p38-MAPK	p38-mitogen-activated protein kinase
PBLs	peripheral blood leucocytes
PBS	phosphate buffer saline
PTPs	protein tyrosine phosphatases
RA	rheumatoid arthritis
RBCs	red blood cells
ROS	reactive oxygen species
SDS	sodium dodecyl sulfate
SDS-PAGE	sodium dodecyl sulfate polyacrylamide gel electrophoresis
SFK	Src family kinase
SH2	domain src homology 2 domain
SHP-2	SH2 domain protein tyrosine phosphatase-2
SLE	systemic lupus erythematosus
Syk	spleen tyrosine kinase
SykI	Syk inhibitors II
TCR	T cell receptor
TNF	tumor necrosis factor
Treg	regulatory t cells
Tyr	tyrosine
