# Supplementary material for: Signaling Response to Transient Redox Stress in Human Isolated T Cells: Molecular Sensor Role of Syk Kinase and Functional Involvement of IL2 Receptor and L-Selectine
Source: Sensors (Basel). 2020 Jan 14;20(2):466. doi: 10.3390/s20020466 (PMC7013990; doi:10.3390/s20020466)
Supplement: Supplementary file 1 [file sensors-20-00466-s001.pdf]

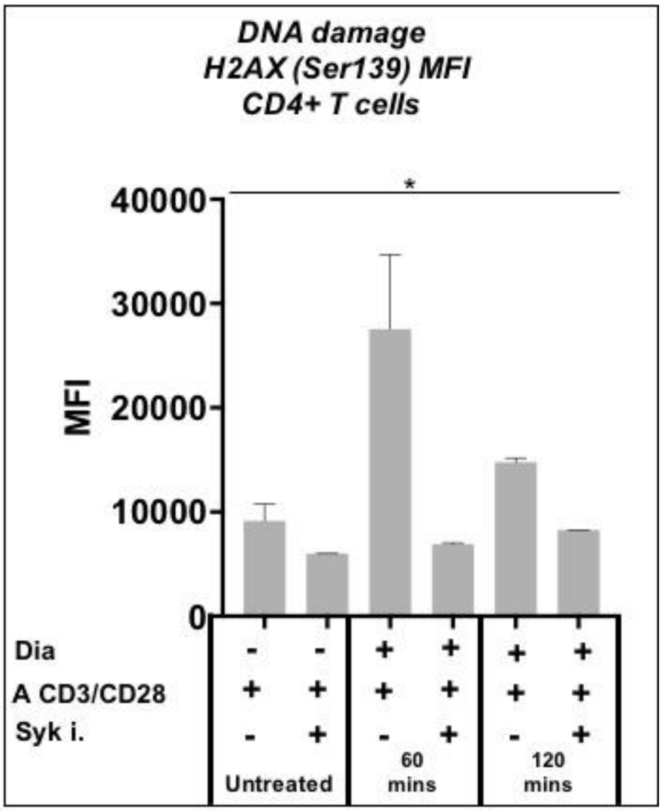

Figure S1. Detection by FACS of histone H2AX phosphorylation on Ser-139. This specific phosphorylation is indicator of DNA damage (DNA double-strand breaks). T cells were treated with/out 0.3 mM diamide in the presence or absence of 5 μM Syk inhibitors (Syk i) at 1-h and 2-h incubation time. T cells were treated with permeabilization buffer (eBioscience, San Diego, CA, USA) and incubated with the mouse anti-human p-Histone H2A.X antibody, and then incubated with the secondary antibody FITC-goat anti-mouse IgG. After incubation, T-cell samples were analyzed by FACS (N = 3). Medians and P values which were found to be statistically significant are shown (* $p < 0.05$). Values are plotted as mean ± error standard.

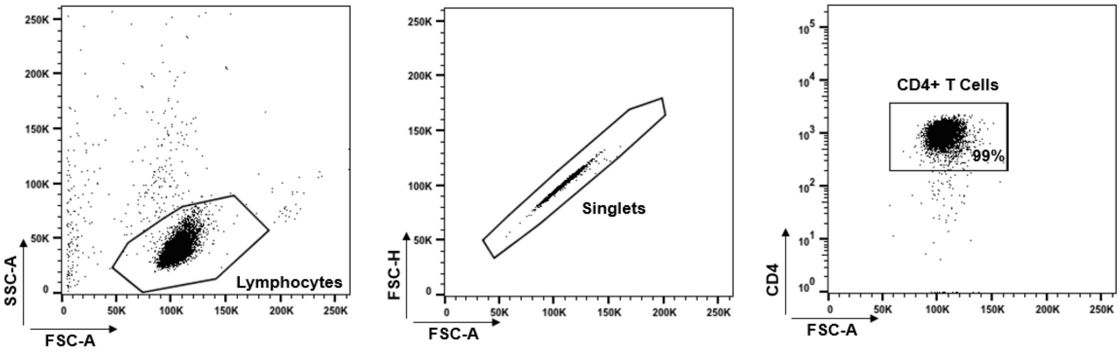

Figure S2. Gating strategy. Gating strategy of negatively isolated CD4+ T cells from human peripheral blood mononuclear cells (PBMCs). The CD4+ T cells purity was always higher than 99% on gated lymphocytes.

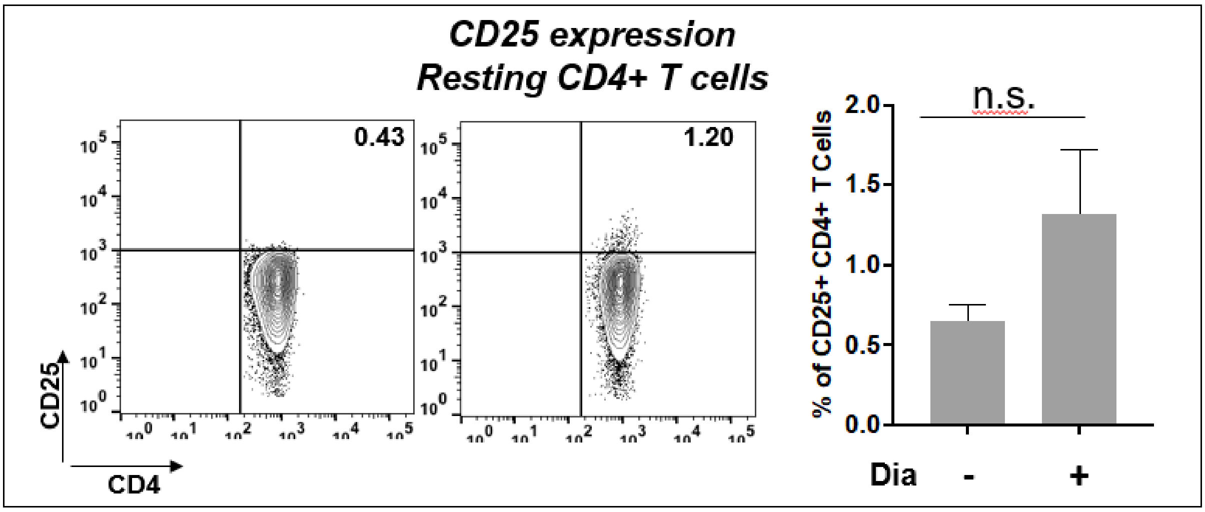

Figure S3. Expression of non-activated T cells CD25 receptor after treatment with diamide. Cells exposed to 0.3mM diamide. Samples were incubated surface-CD25 and analyzed by Flow cytometry (**left** panel): Anti-human CD25 flow representative density plot of untreated and non-activated cells. (**middle** panel): Anti-human CD25 flow representative density plot of non-activated T cells exposed to 0.3 mM diamide. (**right** panel): Medians are showed. P values which were statistically significant are shown (* $p < 0.05$). Values are plotted as mean ± error standard (**A4**). Data are the percentage of total cell population (%).

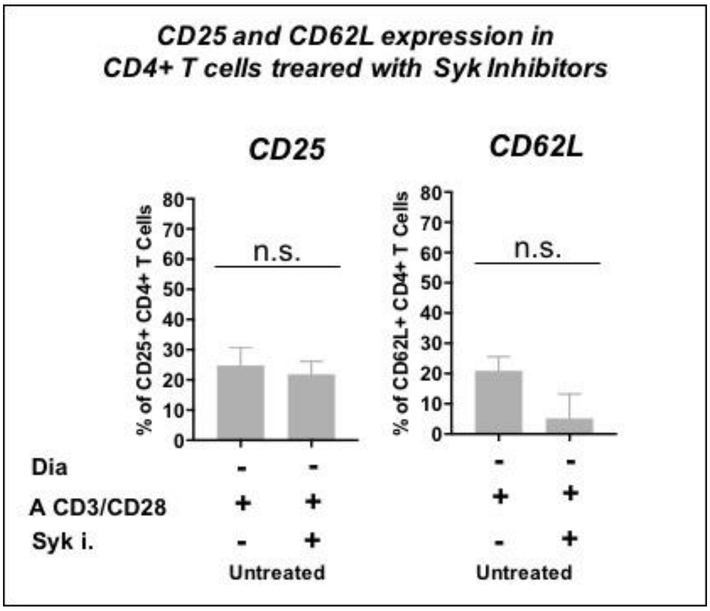

Figure S4. CD25 and CD62L surface receptor expression of T cells treated with Syk inhibitors. T cells were treated with with/out 5 µM Syk inhibitors (Syk i) at 1-h incubation time. T cell samples were stained against surface-CD25 and CD62L and analyzed by FACS (N = 3). Comparison of MFIs between T cell samples in presence versus absence of Syk inhibitors incubation. Values are plotted as mean ± error standard**.** All data are expressed as Median fluorescence intensity (MFI).

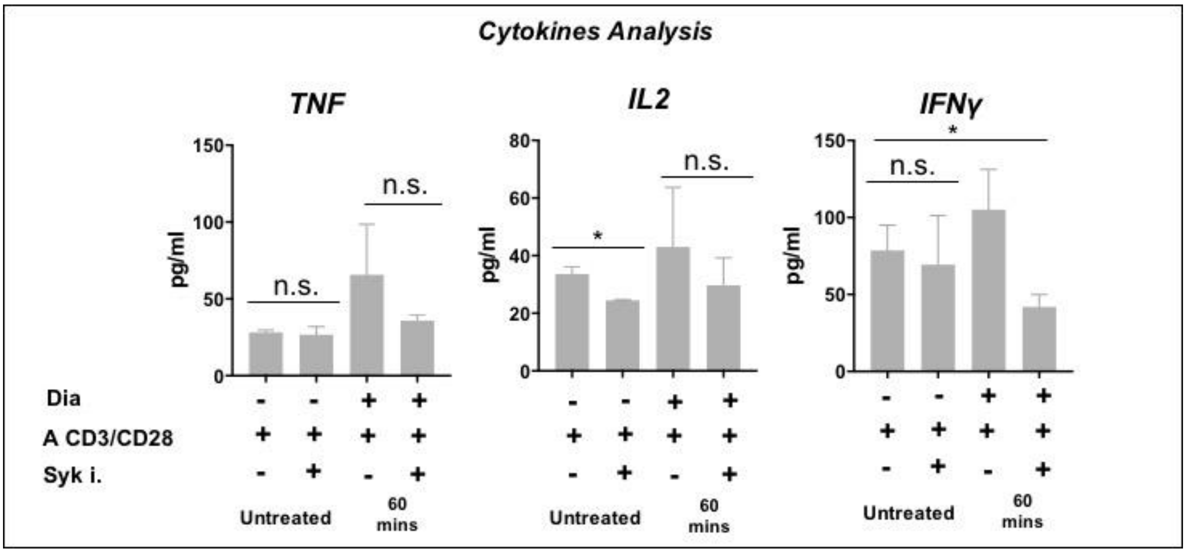

Figure S5. TNFα, IL2, and IFNγ cytokines released by T cells. Cells exposed to 0.3 mM diamide with/out 5 µM Syk inhibitors (Syk i) at 60 minutes incubation time. Cytokines from the supernatants were quantified. Values are showed in w/v picograms (pg/mL) and plotted as mean ± error standard. P values that were statistically significant by the t-student test are shown (* p = 0.05). T cell supernatants were probed against TNFα (left panel) / IL-2 (middle panel) / IFNγ (right panel) by CBA Flex Set method (BD Falcon).
